# Supplementary material for: Predicting visual function by interpreting a neuronal wiring diagram
Source: Nature. 2024 Oct 2;634(8032):113–23. doi: 10.1038/s41586-024-07953-5 (PMC11446822; doi:10.1038/s41586-024-07953-5)
Supplement: Supplementary file 6 — CRF and ERF predictions for individual TmY4 and TmY9 cells. Analogous to Supplementary Data 3, but for TmY target types. Shown are the top four monosynaptic pathways, the strongest pathway passing through each of the top ten intermediary types (ranking from Extended Data Fig. 7), and the trisynaptic pathway Tm1–TmY–Dm3–TmY (see the section entitled Prediction of spatial normalization). [file 41586_2024_7953_MOESM6_ESM.zip › DataS4/TmY9qΓèÑ/720575940627982152.pdf]

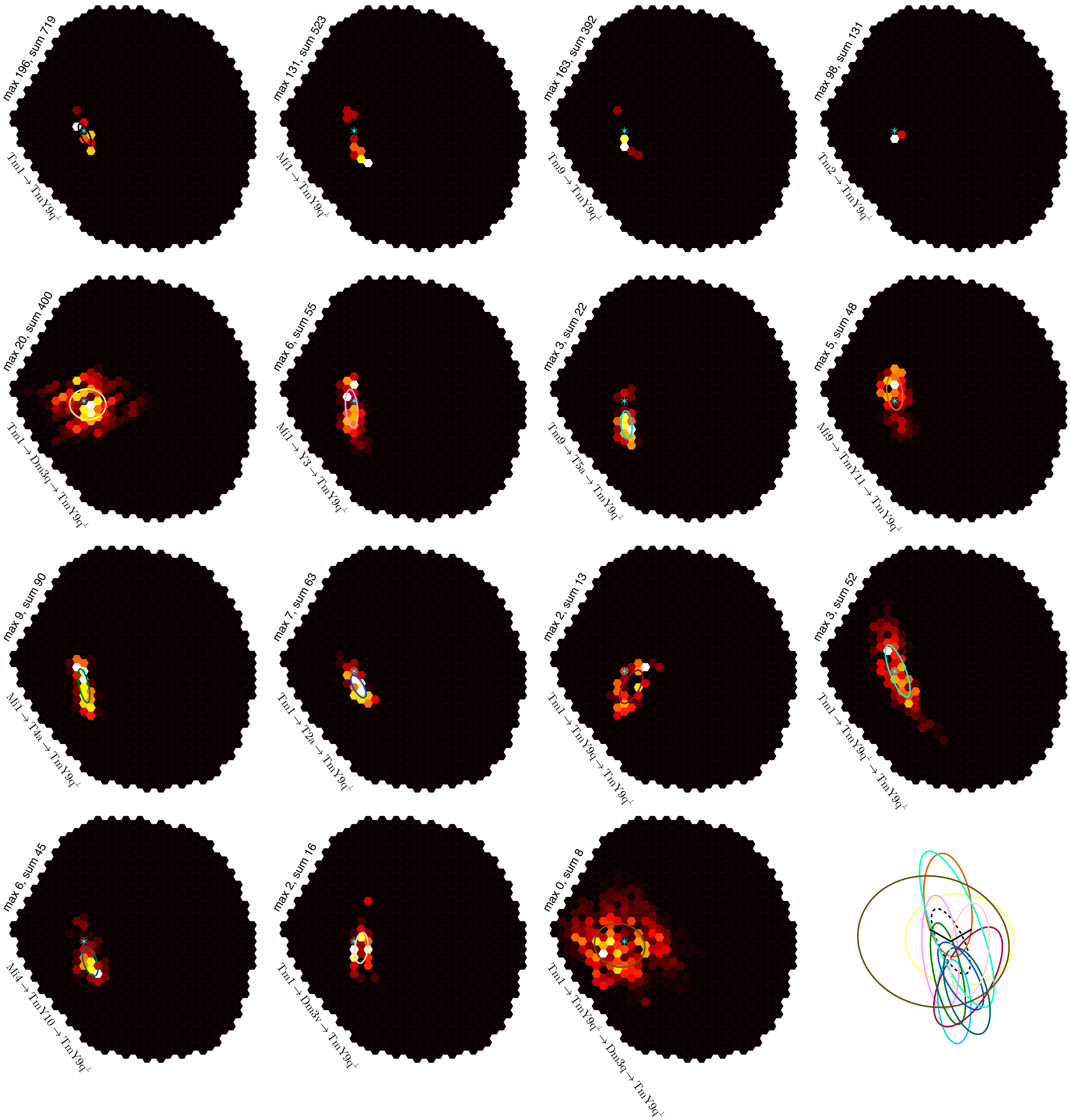

max 196, sum 719
Tm1 → TmY9q⊥
max 131, sum 523
Mi1 → TmY9q⊥
max 163, sum 392
Tm9 → TmY9q⊥
max 98, sum 131
Tm2 → TmY9q⊥
max 20, sum 400
Tm1 → Dm3q → TmY9q⊥
max 6, sum 55
Mi1 → Y3 → TmY9q⊥
max 3, sum 22
Tm9 → T5a → TmY9q⊥
max 5, sum 48
Mi9 → TmY11 → TmY9q⊥
max 9, sum 90
Mi1 → T4a → TmY9q⊥
max 7, sum 63
Tm1 → T2a → TmY9q⊥
max 2, sum 13
Tm1 → TmY9q → TmY9q⊥
max 3, sum 52
Tm1 → TmY9q⊥ → TmY9q⊥
max 6, sum 45
Mi4 → TmY10 → TmY9q⊥
max 2, sum 16
Tm1 → Dm3v → TmY9q⊥
max 0, sum 8
Tm1 → TmY9q⊥ → Dm3q → TmY9q⊥
